# Bodily pleasure matters: velocity of touch modulates body ownership during the rubber hand illusion

**DOI:** 10.3389/fpsyg.2013.00703

**Published:** 2013-10-08

**Authors:** Laura Crucianelli, Nicola K. Metcalf, Aikaterini (Katerina) Fotopoulou, Paul M. Jenkinson

**Affiliations:** ^1^Department of Psychology, University of HertfordshireLondon, UK; ^2^Division of Psychology and Language Sciences, CEHP Research Department, University College LondonLondon, UK

**Keywords:** rubber hand illusion, pleasant touch, interoception, body ownership, embodiment

## Abstract

The sense of body ownership represents a fundamental aspect of our self-consciousness. Influential experimental paradigms, such as the rubber hand illusion (RHI), in which a seen rubber hand is experienced as part of one's body when one's own unseen hand receives congruent tactile stimulation, have extensively examined the role of exteroceptive, multisensory integration on body ownership. However, remarkably, despite the more general current interest in the nature and role of interoception in emotion and consciousness, no study has investigated how the illusion may be affected by interoceptive bodily signals, such as affective touch. Here, we recruited 52 healthy, adult participants and we investigated for the first time, whether applying slow velocity, light tactile stimuli, known to elicit interoceptive feelings of pleasantness, would influence the illusion more than faster, emotionally-neutral, tactile stimuli. We also examined whether seeing another person's hand vs. a rubber hand would reduce the illusion in slow vs. fast stroking conditions, as interoceptive signals are used to represent one's own body from within and it is unclear how they would be integrated with visual signals from another person's hand. We found that slow velocity touch was perceived as more pleasant and it produced higher levels of subjective embodiment during the RHI compared with fast touch. Moreover, this effect applied irrespective of whether the seen hand was a rubber or a confederate's hand. These findings provide support for the idea that affective touch, and more generally interoception, may have a unique contribution to the sense of body ownership, and by implication to our embodied psychological “self.”

## Introduction

The sense of body ownership refers to the feeling that our physical body is our own and a part of our psychological self (Gallagher, 2000). Scientific interest in body ownership has been intense since Botvinick and Cohen (1998) first reported the now well-known rubber hand illusion (RHI), during which participants experience a lifelike rubber hand as part of their body, when their own unseen hand is synchronously stroked. This paradigm is considered one of the few viable ways to experimentally investigate body ownership, because it allows an external object to be subjectively experienced as part of one's body, rather than being simply visually recognized (Tsakiris, 2010). Thus, an abundance of research has sought to reveal the neurocognitive constituents of body ownership during the RHI, revealing that both low-level multisensory integration and high-level body representations contribute to our sense of body ownership (Tsakiris and Haggard, 2005; Makin et al., 2008; Tsakiris, 2010). However, the focus of such studies has mainly been on how the brain integrates different exteroceptive signals, such as vision and touch, to produce the sense of body ownership. Little attention has been paid to how the illusion may be affected by interoceptive bodily signals (defined here as afferent signals that track the physiological state of all tissues of the body, Craig, 2009), such as temperature, pain or pleasant touch. By contrast, in other domains of psychology and cognitive neuroscience, the recent influential discovery of a specialized, interoceptive system that represents the internal, homeostatic state of the body (Craig, 2003) has generated a lot of interest, particularly as regards the scientific study of emotion and self-consciousness. Recent influential accounts of self-awareness link interoception with how we become aware of our body *from within* (Critchley et al., 2004; Craig, 2009; Damasio, 2010; Seth et al., 2012).

In the context of the RHI, Tsakiris et al. (2011) showed that individuals who scored lower in a trait measure of interoceptive sensitivity (heart beat detection task) experienced a stronger RHI compared to individuals who scored higher, possibly reflecting an over-reliance on exteroceptive signals in the former group. It has also been shown that exteroceptive, multisensory integration can have an effect on the physiological regulation of the body during the illusion (Moseley et al., 2008; but see Guterstam et al., 2011). Nevertheless, to our knowledge only one study has attempted to study the reverse relationship, namely what is the specific contribution of interoceptive signals to the illusion and ultimately body ownership. Schütz-Bosbach et al. (2008) found that neither the received (on the subject's own hand), nor the observed (on the seen hand) softness or roughness of tactile stimuli influenced the RHI. However, the degree to which this study activated specific interoceptive pathways is unclear, as it manipulated the materials used to stimulate the hands (cotton vs. sponge), and not the velocity of stroking.

The latter is in fact particularly important for engaging a specialized interoceptive modality, defined as affective, or pleasant, touch. Pleasant touch is coded by specialized, unmyelinated (C-tactile) afferents, found only in hairy skin (Vallbo et al., 1999; Olausson et al., 2002). These afferents respond to slow (between 1 and 10 cm/s), soft touch, and at such velocities the touch on hairy skin is perceived as most pleasant, with a linear correlation between C-tactile firing rates as measured by microneurography and pleasantness ratings on visual-analog scales (Löken et al., 2009). Moreover, C-tactile afferents are distinct from the well-characterized, myelinated tactile fibers that code for discriminative touch (McGlone et al., 2007; Löken et al., 2009). In fact, C-tactile afferents take a distinct ascending pathway from the periphery to the posterior insula (Olausson et al., 2002; Morrison et al., 2011), which is understood to support an early convergence of sensory and affective signals about the body that are then re-represented in the mid and anterior insula, the proposed sites of interoceptive awareness (Critchley et al., 2004; Craig, 2009). Interestingly, the insular cortex has also been linked with the experience of body ownership during the RHI (Tsakiris et al., 2007).

However, the question of how affective touch modulates body ownership in the RHI remains unanswered, as the velocity of tactile stimulation has never been manipulated in previous RHI studies. Moreover, the reporting of the related single velocity procedures in existing RHI studies vary considerably; some authors report only the location and overall duration of stroking (e.g., Maister et al., 2013), while others report the duration of each individual stroke (e.g., Tsakiris and Haggard, 2005), or report no specific details of stroking velocity (e.g., Costantini and Haggard, 2007). Interestingly, in studies reporting velocity details, single frequencies of touch between 1 Hz (Longo et al., 2008; Tsakiris et al., 2011) and 3 Hz (e.g., Bekrater-Bodmann et al., 2012) are typical, corresponding roughly to velocity within the range of pleasant touch. In the current study, we manipulated stroking velocity during the RHI paradigm, by providing light, dynamic tactile stimuli in speeds known to elicit feelings of pleasantness (3 cm/s) vs. speeds known not to elicit such feelings (18 cm/s) (Löken et al., 2009). We predicted that slow velocity stroking would be perceived as more pleasant and lead to greater ownership of the rubber hand than fast velocity stroking.

In addition, while the RHI is not induced when the rubber hand is replaced by a non-corporeal object such as a wooden stick (Tsakiris and Haggard, 2005), it occurs when the rubber hand is replaced by another person's real hand (Schütz-Bosbach et al., 2009). However, it is unclear whether the latter effect would apply when the multisensory integration that underlies the RHI involves integrating vision of another person's hand, with interoceptive signals that are usually used to represent one's own body from within. We thus investigated whether seeing another person's hand vs. a rubber hand would reduce the illusion in slow vs. fast stroking conditions.

## Materials and methods

### Participants

Fifty-two, right-handed women (mean age = 21.04 years, *SD* = 4.05) took part in a single, 45-min testing session. Three participants were later excluded from the data analysis; one did not complete all trials, and two failed to comply with experimental instructions. Institutional ethics approval was obtained and the experiment was conducted in accordance with the Declaration of Helsinki.

### Design and statistical analysis

The experiment used a 2 (Seen Hand: rubber vs. real) × 2 (Stroking Mode: synchronous vs. asynchronous) × 2 (Stroking Velocity: slow vs. fast) mixed factorial design, with repeated measures on the latter two factors (see Table 1). The order of conditions was randomized across participants. For the first, between-subjects manipulation, 24 participants watched a confederate's hand being stroked during the relevant four conditions, whereas 25 participants watched a rubber hand.

**Table 1:** **Table summarizing the experimental design**.

	**Rubber Hand**		**Real Hand**	
	**Slow stroking**	**Fast stroking**	**Slow stroking**	**Fast stroking**
Synchronous	Slow/Synchronous	Fast/Synchronous	Slow/Synchronous	Fast/Synchronous
Asynchronous	Slow/Asynchronous	Fast/Asynchronous	Slow/Asynchronous	Fast/Asynchronous

The dotted line represents the between-subjects factor (Seen Hand), the continuous lines represent the within-subjects factors (Stroking Mode and Stroking Velocity).

Dependent variables comprised: (1) A subjective *pleasantness rating* (7-point Likert-type scale; −3, not at all pleasant; +3, extremely pleasant) of stroking per condition was used to test whether slow touch was perceived as more pleasant than fast touch. (2) An *embodiment questionnaire* (Longo et al., 2008) was used to capture the subjective experience of the illusion (13 statements rated on a 7-point Likert-type scale; −3, strongly disagree; +3, strongly agree). In each condition, the questionnaire was administered pre- (i.e., embodiment due to the visual capture effect) and post-stroking and we calculated their difference to obtain a measure of subjective embodiment due to visuo-tactile integration. This questionnaire consisted of four sub-components: *felt ownership*, that is related to the feeling that the rubber hand was part of one's body; *felt location* of own hand, that related to the feeling that the rubber hand and one's own hand were in the same place; *felt agency*, that is related to the feelings of being able to move the rubber hand; *affect*, that included items related to the experience being interesting, pleasant and enjoyable (Longo et al., 2008). We examined this difference between pre- and post-stroking for each of these components, separately, as well as for an overall “embodiment of rubber hand” (Longo et al., 2008) score, that in our study was obtained by averaging the three subcomponents scores, namely ownership, felt location and felt agency that did not relate to affect. We included this composite measure in order to examine whether the slow touch, which was predicted to be rated as subjectively more pleasant than fast touch, would have an overall effect on aspects of the subjective embodiment of the rubber-hand that were not primarily pleasantness-based. Lastly, we employed (3) a *proprioceptive drift* measure, defined as the degree to which the hand is perceived to be closer to the rubber/real hand after the stroking. In each condition we first subtracted the value corresponding to the *actual* position of the participant's index finger from the value corresponding to the *felt* position (see Procedures below), before (“pre” value) and after (“post” value) stroking and their difference was calculated to obtain a measure of proprioceptive drift due to multisensory integration, as in the case of the embodiment measure explained above. All analyses were conducted using non-parametric tests, as the data were not normally distributed. For confirmatory purposes, the same analyses were also run with parametric tests (ANOVA), revealing the same pattern of findings, but not reported here for brevity.

### Materials

**Figure 1:**
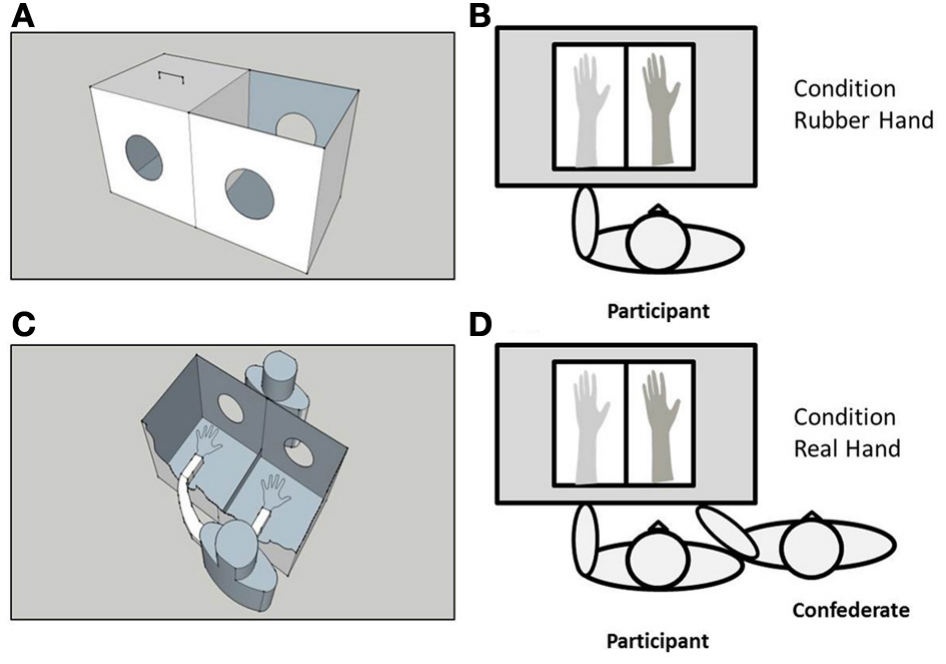
**A schematic representation of the experimental set-up**. A black wooden box measuring 34 × 65 × 44 cm **(A)** was placed approximately 15 cm in front of the participant's torso, with the center of the box in alignment with the participant's left shoulder **(B)**. The box was divided into two equal parts by a perpendicularly placed piece of opaque glass. Two circular holes (14 cm in diameter) on either side of the box allowed the participant and experimenter to place their arms inside; the left half of the box accommodated the participant's left forearm and hand, and the right half the rubber **(C)** or confederate's real **(D)** forearm and hand. A wooden lid (shown in **A**) prevented visual feedback of the participant's own arm. The top side of the box on the right was uncovered, allowing direct vision of the rubber/confederate's forearm and hand.

A black, wooden box measuring 34 × 65 × 44 cm was used to control visual feedback of the participants' arm/hand and the rubber, or the confederate's (real) arm/hand during the experiment (see Figure 1). The box was placed approximately 15 cm in front of the participant's torso, with the center of the box in alignment with the participant's left shoulder. The box was divided into two equal parts by a perpendicularly placed piece of opaque glass. Two circular holes (14 cm in diameter) on either side of the box allowed the participant and experimenter to place their arms inside; the left half of the box accommodated the participant's left forearm and hand, and the right half the rubber, or confederate's forearm and hand. A wooden lid prevented visual feedback of the participant's own arm. The top side of the box on the right was uncovered, allowing direct vision of the rubber/confederate's forearm and hand. The participant also wore a black cape to occlude vision of the proximal end of the rubber/real (confederate) arm and participant's left arm. Tactile stimulation (i.e., stroking) was applied using two, identical, cosmetic make-up brushes (Natural hair Blush Brush, N°7, The Boots Company).

### Procedure

Prior to the main experimental phase, participants were familiarized with procedures and all rating scales. Two adjacent stroking areas, each measuring 9 cm long × 4 cm wide were identified and marked with a washable marker on the hairy skin of participants' left forearm (wrist crease to elbow, McGlone et al., 2012). Stimulation was alternated between these two areas to minimize habituation, and congruent stroking area changes were applied to the rubber/confederate's hand in all instances.

In each condition, the experimenter placed the participant's left hand (palm facing down; fingers pointing forwards) at a fixed point inside the wooden box. A pre-stroking estimate of finger position was obtained using a tailor's tape measure placed on top of the box lid, above the participant's left hand, and in alignment with the coronal (frontal) plane. The section of tape laid across the box was varied across trials to avoid number repetition effects. The participant was asked to report a number on the tape to indicate where they thought their left index finger was located. The experimenter then measured and recorded the actual position of the participant's index left finger. Subsequently, the rubber or the confederate's left arm was positioned in the right half of the box, in front of the participant's body midline, and in the same direction as the participant's actual left arm. The distance between the participant's left arm and the visible arm (on the sagittal plane) was approximately 25 cm. The participant was then instructed to look at the visible arm continuously for 15 s, before completing the pre-stroking *embodiment questionnaire.*

The experimenter then sat opposite the participant and stroked the previously identified stroking areas (McGlone et al., 2012) for 3 min using a speed of either 3 cm/s (slow/pleasant) or 18 cm/s (fast/neutral). In the synchronous conditions, the participant's left forearm and the rubber/confederate's forearm were stroked such that visual and tactile feedback were congruent, whereas in the asynchronous conditions, visual and tactile stimulation were temporally incongruent.

After the stimulation period, the felt and actual location of the participant's left index finger was again measured. Participants then completed the post-stroking *embodiment questionnaire.* Prior to commencing the next condition, they were given a 60 s rest period, during which they were instructed to freely move their left hand.

## Results

### Pleasantness ratings

**Figure 2:**
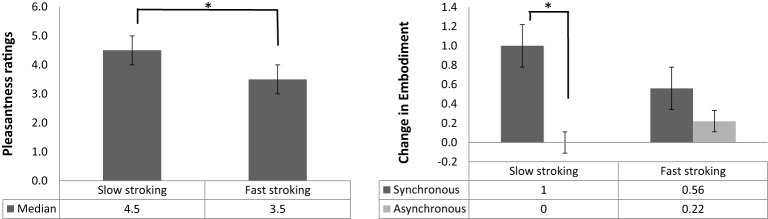
**(A)** Median and interquartile range (error bars) of pleasantness rating scores for slow and fast stroking. **(B)** Median and interquartile range (error bars) of change in embodiment of the rubber/real hand for synchronous (dark gray bars) and asynchronous (light gray bars) stroking, panels **(A,B)**: ^*^p < 0.001.

To establish whether slow stroking was generally perceived by participants as more pleasant than fast stroking, we examined the main effect of Stroking Velocity on pleasantness ratings (Figure 2A). A Wilcoxon signed rank test confirmed that participants rated slow stroking (median = 4.5) as significantly more pleasant than fast stroking (median = 3.5, *Z* = −4.94, *p* < 0.001, *r* = −0.5).

### Embodiment questionnaire—composite score of ownership, location, and agency

#### Main effects

A Wilcoxon signed rank test revealed a main effect of Stroking Mode, with synchronous stroking (median = 0.78) producing significantly higher embodiment scores than asynchronous stroking (median = 0.16; *Z* = −3.44, *p* < 0.001, *r* = −0.35), confirming the classic RHI effect. A Mann–Whitney *U* test on the main effect of Seen Hand revealed that participants embodied the real hand (median = 0.79) to a significantly greater extent than the rubber hand (median = 0.33; *Z* = −2.77, *p* = 0.005, *r* = −0.28). The main effect of Stroking Velocity on embodiment was not significant (*Z* = −1.64, *p* = 0.1, *r* = −0.17).

#### Two-way effects

The interaction between Stroking Mode and Stroking Velocity was analyzed by calculating the difference between synchronous and asynchronous scores in the slow and in the fast stroking conditions separately and subsequently using a Wilcoxon signed rank test to compare these two differential scores. This analysis revealed a significant interaction (*Z* = −3.47, *p* < 0.001, *r* = −0.5). Bonferroni-corrected *post-hoc* analyses (α = 0.025) revealed that, when slow velocity was applied, synchronous stroking resulted in significantly higher embodiment scores compared with asynchronous stroking (*Z* = −4.48, *p* < 0.001, *r* = −0.64, Figure 2B). This comparison was not significant when fast velocity was applied (*Z* = −0.6, *p* = 0.55, *r* = −0.009, Figure 2B). The interaction between Seen Hand and Stroking Mode, as well as the interaction between Seen Hand and Stroking Velocity were likewise analyzed by calculating the relevant differentials and comparing these between groups (real vs. rubber hand) using Mann–Whitney *U* tests. Both interactions were non-significant (*Z* = −0.74, *p* = 0.47, *r* = −0.11 and *Z* = −0.71, *p* = 0.48, *r* = −0.1, respectively).

#### Three-way effects

The interaction between Seen Hand, Stroking Velocity, and Stroking Mode was analyzed by averaging synchronous and asynchronous scores in the slow and the fast stroking conditions separately, calculating their difference, and then analyzing the effect of Seen Hand on this difference using a Mann–Whitney *U* test. This interaction was not significant (*Z* = −0.57, *p* = 0.58, *r* = −0.08).

### Sub-component analysis

#### Main effects

**Figure 3:**
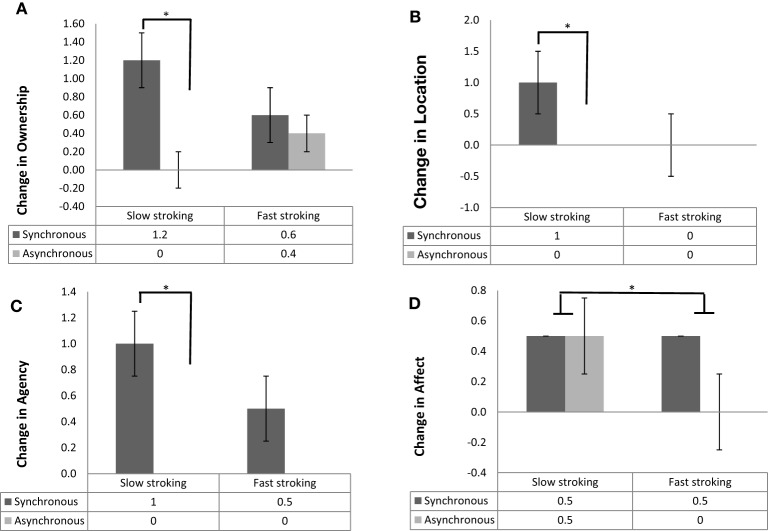
**(A)** Median and interquartile range (error bars) of change in ownership scores for synchrnous (dark gray bars) and asynchronous (light gray bars) stroking. **(B)** Median and interquartile range (error bars) of change in location scores for synchronous (dark gray bars) and asynchronous (light gray bars) stroking. **(C)** Median and interquartile range (error bars) of change in agency scores for synchronous (dark gray bars) and asynchronous (light gray bars) stroking. **(D)** Median and interquartile range (error bars) of change in affect scores for synchronous (dark gray bars) and asynchronous (light gray bars) stroking, panels **(A–C)**: ^*^p <0.001, panel **(D)**: ^*^p = 0.02.

The above analyses were also run on the four subcomponents of the embodiment questionnaire. The pattern of results was identical to the one of the composite embodiment score for the ownership, location and agency subcomponents, while the results for the affect component showed some differences consistent also with the pleasantness ratings results above. Specifically, Wilcoxon signed rank tests revealed a main effect of Stroking Mode, with synchronous stroking producing significantly higher ownership, location, agency and affective component scores than asynchronous stroking (*Z* = −3.55, *p* < 0.001, *r* = −0.36; *Z* = −2.69, *p* = 0.006, *r* = −0.27; *Z* = −3.17, *p* = 0.001, *r* = −0.32; *Z* = −2.38, *p* = 0.02, *r* = −0.24, respectively; Figure 3). However, not surprisingly, there was also a main effect of Stroking Velocity in the affective sub-component, with participants giving significantly higher ratings when slow (median = 0.50) vs. fast (median = 0.25) stroking was applied (*Z*= −2.33, *p* = 0.02, *r* = −0.33, Figure 3D).

#### Two-way effects

The interaction between Stroking Mode and Stroking Velocity was analyzed by calculating the difference between synchronous and asynchronous scores in the slow and in the fast stroking conditions separately and subsequently using a Wilcoxon signed rank test to compare these two differential scores. This analysis conducted separately for the four subcomponents revealed significant interactions for ownership, location and agency (*Z* = −3.27, *p* = 0.001, *r* = −0.33; *Z* = −2.69, *p* = 0.006, *r* = −0.27; *Z* = −2.98, *p* = 0.002, *r* = −0.30, respectively); there was no significant interaction for the affective component (*Z* = −0.098, *p* < 0.9, *r* = −0.01). Bonferroni-corrected *post-hoc* analyses (α = 0.025) revealed that, when slow velocity was applied, synchronous stroking resulted in significantly higher ownership scores (*Z* = −4.43, *p* < 0.001, *r* = −0.45, Figure 3A), location scores (*Z* = −3.43, *p* < 0.001, *r* = −0.35, Figure 3B) and agency scores (*Z* = −3.93, *p* < 0.001, *r* = −0.39, Figure 3C) compared with asynchronous stroking. None of these comparisons was significant when fast velocity was applied (all *p* > 0.12). The interaction between Seen Hand and Stroking Mode, as well as the interaction between Seen Hand and Stroking Velocity were likewise analyzed by calculating the relevant differentials and comparing these between groups (real vs. rubber hand) using Mann–Whitney *U* tests. All interactions were non-significant (all *p* > 0.05).

#### Three-way effects

The interaction between Seen Hand, Stroking Velocity, and Stroking Mode was analyzed by averaging synchronous and asynchronous scores in the slow and the fast stroking conditions separately, calculating their difference, and then analyzing the effect of Seen Hand on this difference using a Mann–Whitney *U* test. This interaction was not significant for any of the subcomponents (*ownership*: *Z* = −0.74, *p* = 0.46, *r* = −0.07; *location*: *Z* = −0.20, *p* = 0.85, *r* = −0.02; *agency: Z* = −1.05, *p* = 0.30, *r* = −0.11; *affective component*: *Z* = −1.50, *p* = 0.13, *r* = −0.15).

### Proprioceptive drift

Proprioceptive drift was analyzed following the same plan of analyses as detailed above. These analyses revealed no significant main effects, two-way effects or three-way effects (all *p*s > 0.10).

## Discussion

Our results confirm previous findings that slow velocity, light touch on hairy skin is perceived as more pleasant than fast touch (Löken et al., 2009). Importantly, we demonstrate for the first time that when such tactile stimulation is congruent to corresponding visual stimuli it produces higher levels of subjective embodiment during the RHI compared with fast, neutral touch. Existing research has examined the effect of various multisensory, exteroceptive signals on embodiment by manipulating factors such as visual-tactile congruency (Botvinick and Cohen, 1998), limb position (Preston, 2013), and physical properties of the materials used to deliver tactile stimulation during the RHI (Schütz-Bosbach et al., 2008). However, to our knowledge, no study to date has specifically examined the effect of engaging the specialized, interoceptive modality of pleasant touch during the RHI. Thus, we provide the first, direct evidence that the perception of specialized interoceptive signals from the skin play an important role in both feelings and judgments of body ownership, as revealed by the different components of the embodiment questionnaire used in the current study. To the extent that the sense of body ownership is considered a fundamental aspect of self-consciousness (Gallagher, 2000), these findings provide support for the idea that interoception lies at the basis of the embodied psychological “self” (Damasio, 1999; Craig, 2009).

Our results further showed that slow, synchronous stroking did not affect the perceived location of the participants' own hand during the illusion. Although classic RHI studies have found that a reliable behavioral measure of the illusion is the degree to which one's arm is felt to be closer in space to the rubber hand (proprioceptive drift, Botvinick and Cohen, 1998; Tsakiris and Haggard, 2005), our finding is consistent with recent studies that showed a dissociation between introspective (embodiment questionnaire) and behavioral (proprioceptive drift) measures of body ownership (Rohde et al., 2011). Our results further specifically suggest that pleasant touch had a greater effect on introspective than behavioral measures of body ownership. This finding thus implies that an interoceptively-mediated embodiment of an external body part does not necessarily involve a spatial update of one's own hand location. This conclusion may also relate to the more general observation that interoceptive pathways mainly convey homeostatic information that are relatively poor in spatial and discriminatory properties in relation to exteroceptive signals (Craig, 2002).

Lastly, our findings showed that participants generally embodied a confederate's hand to a greater extent than a rubber hand, but this difference was unrelated to visuotactile congruency or stroke velocity. Contrary to our prediction, these findings suggest that the top-down knowledge and corresponding visual evidence that one is observing another person's arm, are not sufficient to influence the effect of multisensory integration of congruent visual and tactile signals on body ownership (see also Longo et al., 2009), even if the tactile stimulation carries interoceptive information.

In conclusion, this study shows that dynamic, slow-velocity affective touch can have a fundamental role in the malleability of our sense of body ownership and highlights the central role of interoception and embodied affectivity in self-consciousness. Future studies could determine the precise tactile velocities most likely to maximize the effects of multisensory integration on body ownership, perhaps also in relation to individual differences in pleasant touch perception, as well as more generally interoceptive sensitivity. Furthermore, CT fibers have been reported to innervate only hairy skin (Vallbo et al., 1999) and the majority of RHI studies have applied tactile stroking to hairy skin sites. It would thus be highly interesting to compare in future studies the effects of slow vs. fast stimulation separately on hairy and non-hairy skin sites and examine whether the effects reported in our study would be replicated only in hairy skin sites, as the present findings would suggest. We have also only tested female participants and applied the RHI paradigm only to the left hand, because of the previously reported link of the right insula with interoceptive awareness (Critchley et al., 2004), body ownership and awareness of action (Karnath et al., 2005; Tsakiris et al., 2007; Fotopoulou et al., 2010). Thus, future similar studies should explore the role of gender and right hand stimulation in the observed effect. Moreover, to the extent that pleasant touch (Bermudez, 2005; Björnsdotter et al., 2009) and other interoceptive modalities such as pain (Krahé et al., 2013) are thought to play an essential role in affiliation and social interaction, our findings call for future studies that can investigate the potential role of social, affiliative signals on the sense of body ownership and more generally, the malleability of the bodily self.

## Author contributions

Laura Crucianelli, Aikaterini (Katerina) Fotopoulou, and Paul M. Jenkinson contributed to the concept and design of the study. Testing and data collection were performed by Laura Crucianelli and Nicola K. Metcalf. Laura Crucianelli and Nicola K. Metcalf performed the data analysis and interpretation under the supervision of Aikaterini (Katerina) Fotopoulou and Paul M. Jenkinson. Laura Crucianelli drafted the manuscript, and Aikaterini (Katerina) Fotopoulou and Paul M. Jenkinson provided critical revisions. All authors approved the final version of the manuscript for submission.

### Conflict of interest statement

The authors declare that the research was conducted in the absence of any commercial or financial relationships that could be construed as a potential conflict of interest.
